# Perception-production relationship in children with phonological disorder during speech therapy

**DOI:** 10.1590/2317-1782/e20240246en

**Published:** 2026-03-30

**Authors:** Grazielly Carolyne Ribeiro Fabbro Violanti, Thalia Freitas da Silva, Gabriela Aparecida Rodrigues Gonçalves, Cássio Eduardo Esperandino da Silva, Mayara Ferreira de Assis, Larissa Cristina Berti

**Affiliations:** 1 Departamento Fonoaudiologia, Faculdade de Filosofia e Ciências, Universidade Estadual Paulista Júlio de Mesquita Filho – UNESP - Marília (SP), Brasil.

**Keywords:** Speech, Language and Hearing Sciences, Speech Sound Disorder, Speech Therapy, Speech Perception, Speech Production Measurement

## Abstract

**Purpose:**

To compare and correlate accuracy in perception of others’, self-perception, and speech production in children with phonological disorder during speech therapy.

**Methods:**

Sixteen children participated in a phonological intervention program comprising four stages: perception of the therapist’s speech (perception of others), perception of their own speech (self-perception), and target and probe word production. Performance in each stage was analyzed using percentage accuracy, followed by comparison and correlation analyses.

**Results:**

Performance differed between perceptual skills and speech production. Positive correlations were found between perception of others and target word production, and between self-perception and target and probe word production.

**Conclusion:**

Perceptual accuracy precedes production accuracy. Higher perceptual performance is associated with better speech production outcomes. The correlation between self-perception and untrained words supports targeting this skill to enhance generalization and therapeutic effectiveness.

## INTRODUCTION

Phonological disorder (PD) causes difficulties in acquiring speech sounds during childhood^(1)^, affecting both speech perception and production^(2)^. Researchers initially assumed a direct relationship between these two skills, arguing that perceptual difficulties would necessarily hinder speech production^(3-4)^. However, recent studies indicate that this relationship is not linear. For example, children with typical phonological development may show alterations in speech perception despite adequate production^(5)^, while children with PD may show perceptual performance similar to that of typically developing children even with impaired production^(6,7)^. Thus, high perception scores do not always correspond to high production levels, and perception may be compromised with fully accurate production^(8)^.

Phonological intervention models have traditionally prioritized sound perception in typical adult speech (perception of others), as recommended in models such as the cycles model and its modified version^(9)^, the minimal contrast^(10)^, the maximum opposition^(11)^, the implicational hierarchy of distinctive feature complexity^(12)^, and the ABAB withdrawal^(13)^. Conversely, the perception of the child’s own speech (self-perception) has received little attention, although this skill is fundamental for acquisition^(14-16)^. Furthermore, a recent study^(17)^ found that the relationship between perception and production may vary according to the phonological class analyzed, with a significant correlation observed only for the fricative class.

Therefore, this study examined the relationship between speech production and perception during therapy in children with PD by comparing and correlating three measures: perception of others’ speech, self-perception, and speech production accuracy in children undergoing intervention to suppress the liquid sounds’ substitution.

The focus on liquid substitution reflects its late acquisition and the refined perceptual and articulatory control required by its acoustic-articulatory complexity^(18)^. This process is frequently persistent in children with PD and substantially affects speech intelligibility. Thus, investigating liquid substitution is relevant both clinically, to support more effective interventions, and theoretically, to clarify typical and atypical phonological patterns in Brazilian Portuguese.

The hypotheses were: (1) perception (of others and self) and production skills would differ throughout the intervention, and (2) perception and production skills would show a positive correlation.

## METHOD

### Sample selection

Upon approval by the research ethics committee (CAE no. 30672720.3.0000.5406), we selected 16 children: two from waiting lists of basic health units and 14 enrolled in preschool and elementary levels in nearby public schools. The children were selected by convenience sampling following the inclusion criteria: (a) monolingual children aged 4–8 years (mean age: 5.9), (b) PD diagnosis confirmed by the Child Phonological Assessment, necessarily including—though not limited to—the process of liquid substitution (/ɾ/ → [l] or /l/ → [ɾ])^(18)^, and (c) availability of both children and their guardians to attend the intervention sessions.

Children aged four years and older were included, given that PD can be diagnosed from three years of age, when atypical phonological processes can already be identified. Additionally, children with PD often present multiple speech simplification processes, potentially increasing the risk of delayed phoneme acquisition^(19)^.

Exclusion criteria were: (a) structural alterations of the speech organs (e.g., ankyloglossia, mouth breathing), (b) language disorder diagnosis associated with PD^(1)^; classifications in the public health system follow a medical model, although both conditions involve linguistic alterations in child development, (c) comorbidities affecting speech production, (d) hearing complaints or alterations, defined as auditory thresholds above 15 dBHL at 500, 1000, 2000, and 4000 Hz; (e) neurodevelopmental syndromes or disorders, and (f) non-adherence to the program or risk of withdrawal during the intervention.

After selection and signing of the free and informed consent form, participants underwent speech therapy anamnesis, language assessment in play situations, examination of orofacial structures, basic audiological evaluation, and specific speech assessment using the Speech Assessment Instrument for Acoustic Analysis (IAFAC)^(20)^, which comprises 28 words containing target phonemes in stressed /a/ position.

Speech samples were obtained through repetition and/or spontaneous naming of images and recorded for perceptual-auditory analysis by at least two judges. The analysis included phonetic transcription, phonetic inventory, production variability, phonological system characterization, and PD severity index calculation utilizing the Percentage of Consonants Correct – Revised (PCC-R)^(21)^.

Although repetition and naming rely on different psycholinguistic processes, children with PD frequently avoid naming words during therapy due to the complexity of the required productions. Therefore, therapists encouraged repetition, even when mediated by an adult model. The characterization of the participants, including age, sex, and PD severity (PCC-R)^(21)^, is presented in Table 1. The phonetic-phonological profiles, such as the phonetic inventory (ability to produce speech sounds), the phonological system (development of speech sound representation^(19)^, and phonological processes, are shown in Chart 1.

**Table 1:** The characterization of the study participants

Participant	Age (year)	Sex	PCC-R (%)	Phonological disorder severity
P1	6:5	M	89.7	Mild
P2	5:11	M	87.1	Mild
P3	4:11	F	77.6	Mild-Moderate
P4	7:3	F	91.5	Mild
P5	6:11	M	92.2	Mild
P6	5:5	M	94.2	Mild
P7	4:5	F	62.9	Moderate-Severe
P8	5:0	M	83.5	Mild-Moderate
P9	6:11	M	57.3	Moderate-Severe
P10	6:2	F	82.9	Mild-Moderate
P11	7:0	M	85.3	Mild
P12	8:9	F	82.6	Mild-Moderate
P13	6:3	M	59.0	Moderate-Severe
P14	4:8	M	67.4	Mild-Moderate
P15	4:3	F	73.4	Mild-Moderate
P16	6:7	F	80	Mild

**Caption:** PCC-R = Percentage of Consonants Correct – Revised; M = male; F = female. Source: the authors

**Chart 1:** Phonetic-phonological profiles of the participants

Participant	Phonetic inventory – consonants	Phonological system – effectively acquired	Phonological system – phonemes worked on	Phonological process worked on	Other phonological processes involved
P1	[p, b, t, d, k, g, v, f, s, z, ʒ, ʃ, m, n, ɲ, l, ɾ, x, ɹ]	/p, b, t, d, k, g, v, f, s, z, ʒ, ʃ, m, n, ɲ, x, ɾ, ɹ/	/l/ - not acquired *(50% score)* /ɾ/ - effectively acquired *(100% score)*	Lateral to non-lateral fluid substitution	Gliding of lateral liquid and consonant cluster reduction.
P2	[p, b, t, d, k, g, v, f, s, z, ʒ, ʃ, n, ɲ, l, ɾ, x]	/p, b, t, d, k, g, v, f, s, z, ʒ, ʃ, n, ɲ, x/	/l/ - not acquired *(50% score)* /ɾ/ - not acquired *(0% score)*	Substitution of non-lateral fluid with lateral fluid	Nasal backing, gliding of lateral liquid, retroflex coda deletion, and consonant cluster reduction.
P3	[p, b, t, d, k, g, f, v, s, n, l, x]	/p, t, k, g, s, f, v, s, n, l, x/	/l/ - effectively acquired *(100% score)* /ɾ/ - not acquired *(0% score)*	Substitution of non-lateral fluid with lateral fluid	Obstruent devoicing, fricative fronting, substitution of bilabial nasal with alveolar lateral, palatal nasal deletion, fricative and retroflex coda deletion, gliding of palatal lateral liquid, and consonant cluster reduction.
P4	[p, b, t, d, k, g, v, f, s, z, ʒ, ʃ, m, n, ɲ, l, ʎ, x, ɹ]	/p, b, t, d, k, g, v, f, s, z, ʒ, ʃ, m, n, ɲ, l, x, ɹ/	/l/ - effectively acquired *(100% score)* /ɾ/ - not acquired *(0% score)*	Substitution of non-lateral fluid with lateral fluid	Consonant cluster reduction and substitution.
P5	[p, b, t, d, k, g, v, f, s, z, ʒ, ʃ, m, n, ɲ, l, ʎ, ɾ, x, ɹ]	/p, b, t, d, k, g, v, s, z, ʒ, ʃ, m, n, ɲ, l, ʎ, x, ɹ/	/l/ - effectively acquired *(100% score)* /ɾ/ - not acquired *(0% score)*	Substitution of non-lateral liquid with lateral liquid and vice versa.	Substitution of non-lateral consonant cluster with lateral.
P6	[p, b, t, d, k, g, v, f, s, z, ʒ, ʃ, m, n, ɲ, l, ʎ, x, ɹ]	/p, b, t, d, k, g, v, f, s, z, ʒ, ʃ, m, n, ɲ, l, ʎ, x, ɹ/	/l/ - effectively acquired *(100% score)* /ɾ/ - not acquired *(0% score)*	Substitution of non-lateral fluid with lateral fluid	Consonant cluster reduction and substitution.
P7	[p, b, t, d, k, g, f, v, s, ʃ, ʒ, m, n, ɲ, l, x]	/p, b, t, d, v, s, ʃ, ʒ, m, n, ɲ, l, x/	/l/ - effectively acquired *(100% score)* /ɾ/ - not acquired *(0% score)*	Substitution of non-lateral fluid with lateral fluid	Plosive fronting, fricative backing, substitution of lateral liquid with alveolar, gliding of retroflex coda, and substitution of non-lateral consonant cluster with lateral.
P8	[p, b, t, d, k, g, f, v, s, z, ʒ, m, n, ɲ, l, ɾ, x]	/p, b, t, d, k, g, f, v, s, z, ʒ, m, n, ɲ, x/	/l/ - not acquired *(33.3% score)* /ɾ/ - emerging *(55.5% score)*	Substitution of non-lateral liquid with lateral liquid and vice versa.	Fricative fronting, liquid deletion and gliding, retroflex coda deletion, and consonant cluster reduction.
P9	[p, b, t, k, f, ʃ, ʒ, m, n, ɲ, l, ɾ, x]	/p, t, k, ʃ, m, n, ɲ, x/	/l/ - not acquired *(50% score)* /ɾ/ - not acquired *(12% score)*	Substitution of non-lateral liquid with lateral liquid and vice versa.	Obstruent devoicing, plosivization and backing of fricative, deletion of semivowel, retroflex, and nasal codas, substitution of lateral liquid, and consonant cluster reduction.
P10	[p, b, t, d, k, g, f, v, s, z, m, n, ɲ, l, ʎ, x]	/p, b, t, d, k, g, f, v, s, z, m, n, ɲ, l, ʎ, x/	/l/ - effectively acquired *(100% score)* /ɾ/ - not acquired *(0% score)*	Substitution of non-lateral fluid with lateral fluid	Fricative fronting, gliding of retroflex coda, and consonant cluster reduction.
P11	[p, b, t, d, k, g, f, v, s, z, ʃ, ʒ, m, n, ɲ, l, ʎ, x, ɹ]	/p, b, t, d, k, g, f, v, s, z, ʃ, ʒ, m, n, ɲ, l, ʎ, x, ɹ/	/l/ - effectively acquired *(100% score)* /ɾ/ - not acquired *(0% score)*	Substitution of non-lateral fluid with lateral fluid	Consonant cluster reduction.
P12	[p, b, t, d, k, g, f, v, s, z, ʃ, ʒ, m, n, ɲ, l, ʎ, x]	/p, b, t, d, k, g, f, v, s, z, ʃ, ʒ, m, n, ɲ, l, ʎ, x/	/l/ - effectively acquired *(100% score)* /ɾ/ - not acquired *(0% score)*	Substitution of non-lateral fluid with lateral fluid	Retroflex coda deletion and consonant cluster reduction.
P13	[p, b, t, k, g, f, v, s, ʒ, m, ɲ, l, x]	/p, b, k, g, f, v, m, ɲ, x/	/l/ - not acquired *(50% score)* /ɾ/ - not acquired *(0% score)*	Substitution of non-lateral fluid with lateral fluid	Backing of obstruents and alveolar nasals, deletion of lateral and non-lateral liquids, gliding of palatal lateral liquid, retroflex coda deletion, and consonant cluster reduction.
P14	[p, b, t, d, k, g, f, v, s, ʃ, m, n, ɲ, l, x]	/p, b, d, k, g, s, n, ɲ, l, x/	/l/ - effectively acquired *(100% score)* /ɾ/ - not acquired *(0% score)*	Substitution of non-lateral fluid with lateral fluid	Obstruent backing, substitution of labiodental fricative with alveolar, substitution of bilabial nasal with lateral liquid, fricative devoicing, plosivization of alveolar fricative into velar stop, substitution of palatal liquid with dentoalveolar liquid, fricative and retroflex coda deletion, substitution of plosive with fricative, metathesis in consonant cluster, and consonant cluster reduction.
P15	[p, b, t, d, k, g, f, v, s, ʃ, m, n, ɲ, l, ʎ, ɾ, x, ɹ]	/p, b, t, d, k, g, f, ʃ, m, n, ɲ, l, ʎ, x/	/l/ - effectively acquired *(100% score)* /ɾ/ - not acquired *(0% score)*	Substitution of non-lateral fluid with lateral fluid	Plosivization, fronting and devoicing of fricative, gliding of retroflex coda, and consonant cluster reduction.
P16	[p, b, t, d, k, g, f, v, s, z, ʃ, ʒ, m, n, ɲ, l, ʎ, x, ɹ]	/p, b, t, d, f, v, s, z, ʃ, ʒ, m, n, ɲ, l, ʎ, x, ɹ /	/l/ - effectively acquired *(100% score)* /ɾ/ - not acquired *(30% score)*	Substitution of non-lateral fluid with lateral fluid	Plosivization and fronting of fricative, gliding of retroflex coda, fricative devoicing, and consonant cluster reduction.

Source: the authors

### Intervention program

The phonological intervention program was based on the perception-production model (Berti, unpublished results), currently under development and validation. This is the only model that simultaneously addresses the therapist’s perception of the child’s speech (perception in others), the child’s perception of their own speech (self-perception), and speech production.

The intervention comprised 16 speech-therapy sessions targeting the phonological process of liquid sound substitution (/l/ → [ɾ] ou /ɾ/ → [l]). Sessions were conducted on Tuesdays and Fridays, lasted 50 minutes each, and resulting in 13 hours and 30 minutes of treatment. The number of sessions followed the literature recommendations of a minimum of 15 therapy sessions^(2)^. Children completed treatment before the 16th session if the phonological process was overcome, or continued to receive care at the health service when further intervention was required.

Thirty target words (with the target sounds /l/ and /ɾ/ in simple onset position) were selected for all intervention stages, and another 30 probe words (with the same target sounds in simple onset) were used exclusively to assess generalization. Most words formed minimal or analogous pairs, as presented in Table 2.

**Table 2:** Target and probe words presented to the participants

Target words		Probe words	
/ɾ/	/l/	/ɾ/	/l/
Vera	Vela	Vareta	Valeta
Mara	Mala	Carango	Calango
Caro	Calo	Barão	Balão
Puro	Pulo	Pário	Palio
Cara	Cala	Caruso	Caluso
Sara	Sala	Peru	Pelu
Vira	Vila	Gari	Gali
Vara	Vala	Poro	Pólo
Corado	Colado	Rara	Rala
Cera	Sela	Garo	Galo
Mora	Mola	Gera	Pala
Marinha	Malinha	Farinha	Bala
Mira	Mila	Jararaca	Cola
Sarada	Salada	Maracá	Bela
Coragem	Colagem	Pera	Pistola

Source: the authors

The intervention was structured in six stages:

1. Initial assessment: Collection and analysis of speech samples using the IAFAC^(20)^ for participant selection and intervention initiation.
2. Pre-therapy: Initial collection with audio recording of spontaneous naming or repetition of the 30 target and 30 probe words, presented via computer images and contextualized with playful activities and toys. Productions were recorded with minimal therapist interference.
3. Phonological process explanation: Contrastive introduction of target sounds (/l/ and /ɾ/) using playful materials (e.g., mouth and tongue models built from recyclable materials) to demonstrate tongue movements (rapid elevation and tapping of the tongue producing [ɾ] and slow elevation and tapping of the tongue producing [l]). Minimal pairs of target words were used to illustrate how changing sounds can alter word meaning (e.g., [´kaɾu] ‘expensive’ vs. [´kalu] ‘callus’).
4. Perception of others: The therapist produced the 30 target words live and naturally, asking the children to identify the target sound ([l] or [ɾ]) during games of interest. A target word would be randomly selected, and the therapist would position themself in front of and at the child’s eye level, directing the child’s attention to their face. They then produced the word slowly with emphasis on the target sound, and the child identified the sound produced. Responses were recorded as correct or incorrect.
5. Self-perception: Children produced the 30 target words and then reported which target sound they had produced ([l] or [ɾ]). Auditory, visual, and/or proprioceptive cues were provided as needed, and responses were recorded as correct or incorrect.
6. Production: Children produced the 30 target words. When necessary, visual aids (e.g., use of a mirror) and tactile-kinesthetic cues (e.g., repeated production of [d] or [l]) were utilized to facilitate target production, always in a playful and engaging context. Productions were recorded as correct, incorrect, or graded (intermediate achievements between incorrect and correct).
7. Post-therapy: Final data collection, reapplication of the IAFAC^(20)^, and recording the naming or repetition of the 30 target and 30 probe words under conditions identical to those of the initial assessment.

**Figure 1:**
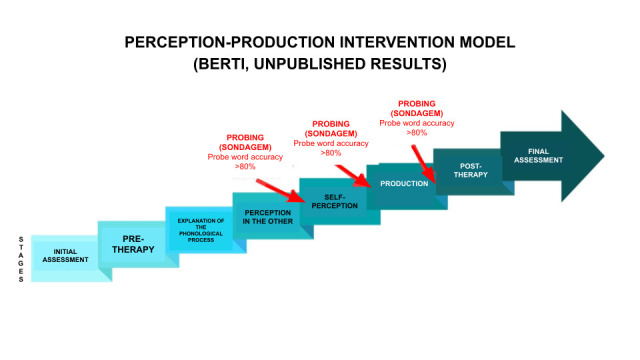
Proposed speech-language therapy intervention model

Between the stages of perception of others, self-perception, and production, children were assessed with words not worked on in therapy. Advancement to the next stage required achieving 80% correct responses, following the criteria of Yavas et al.^(17)^, who consider a contrastive sound acquired at 76–85% accuracy. The intervention scheme is shown in Figure 1.

All intervention stages were conducted by two trained speech therapists and master’s students in Speech Therapy, assisted by a supervised undergraduate speech therapy student. Recordings of target and probe word productions were collected in every session, resulting in 512 recordings (one recording of each target and probe word per session × 16 sessions × 16 children). Productions were evaluated by two speech-language pathologists who classified the sounds produced as (1) correct, (2) incorrect, or (3) graded. Disagreements were resolved by a third speech-language pathologist, who made the final decision. Subsequently, the percentage of correct responses over the sessions was calculated.

### Statistical analysis

For inferential statistical analysis, the children’s accuracy was assessed at six therapeutic moments: (1) pre-therapy target word production; (2) pre-therapy probe word production; (3) perception of others in the first session; (4) self-perception in the first session; (5) post-therapy target word production; and (6) post-therapy probe word production. Comparative analysis used percentage performance (0–100% correct) for each stage. For correlation analysis, individual responses for each word (30 target and 30 probe words) were used, categorized as (1) correct or (0) error, for both perception and production. Repeated measures ANOVA was employed to compare performances, followed by Tukey’s post-hoc test to identify differences between skills. For correlation analysis, Spearman’s nonparametric correlation test was used to evaluate the strength of association between variables, with coefficients ranging from –1 to +1 (where 0 indicates no correlation and ±1 indicates perfect correlation). The significance level was set at α < 0.05.

## RESULTS

**Figure 2:**
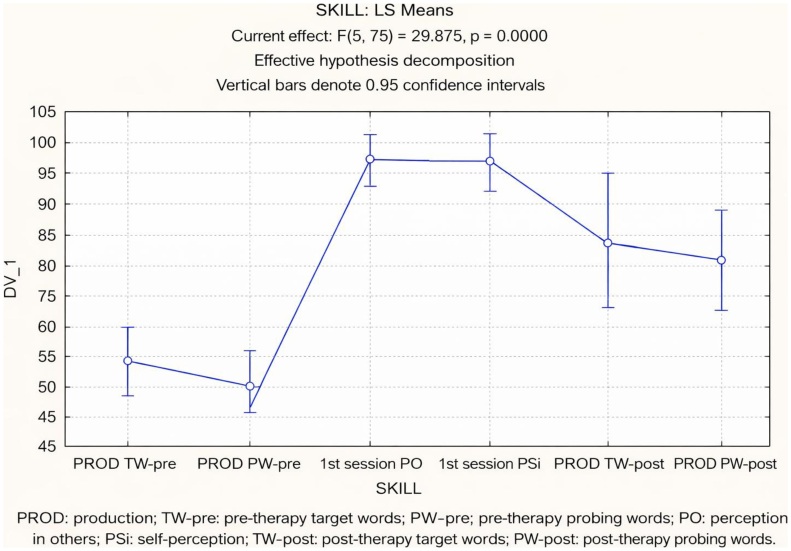
Illustration of the effect between abilities (repeated measures ANOVA)

The skills’ accuracy throughout the intervention, expressed as mean percentage correct and standard deviation, is presented in Table 3. It was examined across target and probe word production in pre-therapy, perception of others, and self-perception in the first session, and target and probe word production in post-therapy. The results of the repeated measures ANOVA are shown in Table 4. A statistically significant difference was observed among the skills [F(5,25) = 29.87, p < 0.01]. Tukey’s post hoc test revealed significant differences between target and probe word production at both the beginning and the end of the intervention, as well as between perception skills (perception of others and self-perception) and production skills (target and probe word production). No significant differences were found between perception of others and self-perception, nor between target and probe word production in pre-therapy. Children’s accuracy across the therapeutic process is illustrated in Figure 2.

**Table 3:** Mean percentage of correct answers for the skills worked on in the intervention process

Participant	Pre-therapy TW production	Pre-therapy PW production	1^st^ session PO	1^st^ session PSi	Post-therapy TW production	Post-therapy PW production
P1	60	50	96.6	100	96.6	90
P2	50	59.2	100	70	100	80
P3	50	50	94.7	93.3	50	60
P4	53.3	50	80	93.3	50	60
P5	63.3	70	100	96.6	96.6	93.3
P6	63.3	70	100	96.6	96.6	90
P7	50	50	89.6	96.6	50	56.6
P8	66.6	53.3	80	83.3	56.6	56.6
P9	70	70	86.6	83.3	76.6	63.3
P10	50	50	76.6	93.3	96.6	76.6
P11	66.6	60	96.6	100	83.3	76.6
P12	56.6	53.3	100	100	83.3	86.6
P13	23.3	20	100	100	20	36.6
P14	60	50	93.3	83.3	63.3	63.3
P15	50	53.3	90	100	75.8	83.3
P16	86.6	60	96.6	93.3	100	90
Overall mean	57.5	54.3	92.5	92.7	74.7	72.7
Standard deviation	13.46	11.89	7.97	8.54	23.99	16.40

**Caption:** TW = Target words; PW = probe words; PO = perception in the other; PSi = self-perception; S = subject. Source: the authors

**Table 4:** Comparison between abilities (repeated measures ANOVA)

Skills	Pre-therapy TW production	Pre-therapy PW production	1^st^ session PO	1^st^ session PSi	Post-therapy TW production	Post-therapy PW production
Pre-therapy TW production		0.9617	**0.0001** *	0.0001*	0.0002*	0.0009*
Pre-therapy PW production	-		0.0001*	0.0001*	0.0001*	0.0001*
1^st^ session PO	**-**	**-**		1.0000	0.0134*	0.0023*
1^st^ session PSi	**-**	**-**	-		0.0016*	0.0028*
Post-therapy TW production	**-**	**-**		**-**		0.9929
Post-therapy PW production	**-**	**-**	**-**	**-**		

* Bold values represent p < 0.05

**Caption:** TW = Target words; PW = probe words; PO = perception in the other; PSi = self-perception; S = subject. Source: the authors

**Caption:** PROD = production, TW-pre = pre-therapy target words; PW-pre = pre-therapy probing words; PO = perception in others; PSi = self-perception; TW-post = post-therapy target words; PW-post = post-therapy probing words. Source: the authors

The correlation data among perception of others, self-perception, target word production, and probe word production are presented in Table 5. Positive correlations were identified between perception of others and post-therapy target word production (r = 0.11), self-perception and post-therapy target word production (r = 0.08), and self-perception and post-therapy probe word production (r = 0.10). The strongest correlation was observed between perception of others and target word production, suggesting that initial perceptual performance may directly influence improvement in word production during therapy.

**Table 5:** Direction and strength of correlation between skills (r-value) (Spearman’s correlation test)

Variables	Pre-therapy TW production	Pre-therapy PW production	1^st^ session PO	1^st^ session PSi	Post-therapy TW production	Post-therapy PW production
Pre-therapy TW production		**0.182** *	0.036	-0.039	0.280*	0.078
Pre-therapy PW production	**-**		-0.008	-0.030	-0.082	0.377*
1^st^ session PO	-	-		0.034	0.119*	0.059
1^st^ session PSi	-	-	-		0.085*	0.106*
Post-therapy TW production	**-**	-	**-**	**-**		0.269*
Post-therapy PW production	-	**-**	-	**-**	**-**	

* Bold values represent p < 0.05

**Caption:** TW = Target words; PW = probe words; PO = perception in the other; PSi = self-perception; S = subject. Source: the authors

To clarify the generalization observed at the end of the intervention, the percentage of correct responses to probe words in post-therapy, by participant, is shown in Chart 2. Children with mild PD generally achieved higher rates of correct responses for untrained words, reflecting greater generalization. In contrast, participants with moderate to severe PD mostly had lower percentages, pointing to a possible association between disorder severity and generalization ability.

**Chart 2:** Percentage scores on the probe words at the end of the intervention (generalization indicator)

Participant	PCC-R (%)	PD severity	PW accuracy after therapy (%)
P1	89.7	Mild	90
P2	87.1	Mild	80
P3	77.6	Mild-Moderate	60
P4	91.5	Mild	60
P5	92.2	Mild	93.3
P6	94.2	Mild	90
P7	62.9	Moderate-Severe	56.6
P8	83.5	Mild-Moderate	56.6
P9	57.3	Moderate-Severe	63.3
P10	82.9	Mild-Moderate	76.6
P11	85.3	Mild	76.6
P12	82.6	Mild-Moderate	96.6
P13	59.0	Moderate-Severe	36.6
P14	67.4	Mild-Moderate	63.3
P15	73.4	Mild-Moderate	83.3
P16	80	Mild	90

**Caption:** PW = Probe words; PCC-R = Percentage of Consonants Correct - Revised; PD = phonological disorder. Source: the authors based on Table 2

Source: the authors

Overall, the findings confirmed that the 16 children performed differently during the intervention, underscoring the clinical heterogeneity of PD. Comparison of the skills revealed variations between perception and production, while the observed correlations support the positive influence of perceptual improvement on speech production. Furthermore, correct probe word production at the end of the intervention demonstrates that self-perception may be associated with the target sound generalization, with superior performance in children with mild PD.

## DISCUSSION

This study aimed to compare and correlate the accuracy of perception in others, self-perception, and word production in children with PD receiving speech therapy. The first hypothesis predicted that perception and production skills would exhibit distinct performance patterns during intervention. When accuracy was compared across perception of others, self-perception, and target and probe word production (pre- and post-therapy), statistically significant differences were observed, confirming this hypothesis. However, no significant difference emerged between the perception of others and self-perception, indicating that these abilities share similarities.

This similarity likely results from both skills requiring the child’s awareness of the target sounds (/l/ and /ɾ/)^(21)^. Although self-perception is recognized as essential for phonological acquisition, it has been underexplored in the literature^(19-22)^. Various therapeutic models^(9-13)^ emphasize auditory perception based on typical adult speech, often to the detriment of attentive listening to the child’s own productions. To date, no studies have directly compared the perception of others’ with self-perception during therapy, albeit research such as Battistela^(23)^ has highlighted its importance.

The findings also demonstrated that perception and production follow distinct developmental paths during therapy, supporting previous evidence that accurate perception does not necessarily lead to adequate production^(5-7)^. In the present study, seven of the sixteen children had not acquired the target phonemes by the end of the intervention. This outcome may relate to their younger age, a larger number of phonological processes, and/or a more restricted phonetic inventory.

The quantitative improvement in the accuracy of target and probe word production at the pre-therapy stage, as well as in perception of others and self-perception, was potentially influenced by the phonological process explanation. At this stage, the target sounds (/l/ and /ɾ/) were contrasted through visual and playful resources that illustrated the articulatory movements required for each production. This preliminary work appears to have fostered greater articulatory awareness, improving both the perceptual sound recognition and the children’s conscious identification of their own productions. This phenomenon may have supported development in perception and, indirectly, in production.

The diversity in individual responses highlights how differently PD can manifest, pointing to future studies that should more carefully consider factors such as age, disorder severity, and each child’s phonetic-phonological profile. Additionally, when children’s performance was examined according to PD severity, as measured by the revised percentage of correct consonants (PCC-R)^(21)^, those with mild PD tended to present higher accuracy in both perception and production than those with moderate or severe PD. These results suggest that disorder severity may influence intervention response, especially regarding generalization to untrained words. Nevertheless, given the limited sample size, these findings should be interpreted with caution and warrant further investigation.

The second hypothesis predicted a positive correlation between speech perception and production skills and was also confirmed. Positive correlations were found between perception of others and target word production, as well as between self-perception and target and probe word production. This relationship corroborates that improved perception of others corresponds to better production of therapy-targeted words. Higher self-perception performance also corresponds to better production of both targeted and untrained words, emphasizing the relevance of self-perception for generalization.

Indeed, higher accuracy in probe words at the end of the intervention supports that children who performed better during self-perception also exhibited higher generalization rates for untrained words. Moreover, participants with mild PD achieved a greater number of correct responses. Thus, disorder severity appears to influence the effectiveness of skill generalization, which is consistent with previous research demonstrating an association between perceptual performance, PD severity, and therapy success in untrained contexts^(24)^. These correlations between perception and production show that perception should be addressed before speech production in phonological intervention, as recommended in phonological intervention approaches^(9-13)^.

The strongest correlation was observed between perception in others and post-therapy target word production (r = 0.12), followed by the correlation between self-perception and probe word production (r = 0.10), indicating that both skills should be explored complementarily. Perception of others encompasses more stable stimuli with robust acoustic cues, which can facilitate contrast recognition. Conversely, self-perception requires children to analyze their own, often still inaccurate^(16)^, productions, thereby increasing task difficulty while remaining potentially beneficial in the long term.

The relationship between perception and production is complex and warrants further investigation in studies with larger samples. Future research should also consider PD severity, socioeconomic and socioeducational factors, and the influence of other phonological processes, as these aspects may elucidate the perception-production relationship during therapy.

## CONCLUSION

During speech therapy intervention, children with PD exhibited stronger performance in perception (in others and self-perception) than in speech production. Positive correlations emerged between perception in others and target word production, and between self-perception and target and probe word production. These findings suggest that improvements in perception can facilitate speech production. Self-perception was associated with target generalization (/l/ and /ɾ/) to trained and untrained sounds. Therefore, this study supports the inclusion of activities targeting the child’s own speech perception in therapy planning to promote a more effective treatment.
