# Supplementary material for: Perception-production relationship in children with phonological disorder during speech therapy
Source: Codas. 2026 Mar 30;38(2):e20240246. doi: 10.1590/2317-1782/e20240246en (PMC13075836; doi:10.1590/2317-1782/e20240246en)
Supplement: Translation

# Translation: Relação entre percepção e produção de fala em crianças com transtorno fonológico no processo de intervenção fonoaudiológica

**DOI:** 10.1590/2317-1782/e20240246pt

**Authors:** Grazielly Carolyne Ribeiro Fabbro Violanti, Thalia Freitas da Silva, Gabriela Aparecida Rodrigues Gonçalves, Cássio Eduardo Esperandino da Silva, Mayara Ferreira de Assis, Larissa Cristina Berti

**Affiliations:** 1 Departamento Fonoaudiologia, Faculdade de Filosofia e Ciências, Universidade Estadual Paulista Júlio de Mesquita Filho – UNESP - Marília (SP), Brasil.

**Keywords:** Fonoaudiologia, Transtorno Fonológico, Fonoterapia, Percepção da Fala, Medida da Produção da Fala

## Abstract

**Objetivo:**

Comparar e correlacionar as acurácias na percepção no outro, percepção em si e produção de fala em crianças com Transtorno Fonológico no processo de intervenção fonoaudiológica.

**Método:**

Participaram do estudo 16 crianças com Transtorno Fonológico, submetidas a um programa de intervenção fonológica que contemplava as seguintes etapas: percepção da fala do terapeuta (percepção no outro), percepção da própria fala (percepção em si), produção de fala em palavras trabalhadas (palavras-alvos) e produção de fala em palavras não trabalhadas (palavras-sondagem). O desempenho dos sujeitos em cada etapa foi analisado a partir da porcentagem de acerto (acurácia), possibilitando a análise de comparação e correlação.

**Resultados:**

Na análise de comparação, os desempenhos das crianças mostraram-se distintos entre as habilidades de percepção (no outro e em si) e produção de fala. Na análise de correlação, observou-se correlação positiva entre percepção no outro e produção de palavras trabalhadas e entre percepção em si e produção de palavras trabalhadas e não trabalhadas.

**Conclusão:**

A acurácia na percepção antecede a acurácia na produção. Quanto melhor o desempenho na percepção, melhor será o desempenho na produção de palavras em terapia. A correlação positiva entre o desempenho na percepção em si a produção de palavras não trabalhadas sugere que esta habilidade deva ser trabalhada com vistas à generalização e, consequentemente, uma maior eficácia terapêutica.

## INTRODUÇÃO

O Transtorno Fonológico (TF) é caracterizado por dificuldades na aquisição dos sons da fala durante a infância^(1)^, afetando tanto a percepção quanto a produção da fala^(2)^.

Inicialmente, acreditava-se que havia uma relação direta entre essas duas habilidades — ou seja, dificuldades na percepção levariam automaticamente às dificuldades na produção^(3-4)^. No entanto, estudos mais recentes indicam que essa relação não é linear. Ou seja, crianças com desenvolvimento fonológico típico podem apresentar alterações na percepção da fala, mesmo com produções adequadas^(5)^; ou crianças com TF podem ter desempenho perceptivo semelhante ao de crianças típicas, ainda que suas produções estejam comprometidas^(6-7)^. Dessa forma, escores elevados de percepção nem sempre correspondem a níveis altos de produção, e é possível que a percepção esteja prejudicada mesmo diante de 100% de acerto de produção^(8)^.

Os modelos de intervenção fonológica têm priorizado a percepção dos sons na fala do adulto típico (percepção no outro), tal como preconiza os seguintes modelos: o modelo de ciclos e sua versão modificada^(9)^, o modelo de pares mínimos^(10)^, o modelo de oposições máximas^(11)^, o modelo de hierarquia implicacional de complexidade de traços distintivos^(12)^ e o modelo ABAB retirada^(13)^. No entanto, a percepção da própria fala da criança (percepção em si) tem sido pouco explorada, embora o trabalho com essa habilidade possa ter papel relevante na aquisição fonológica^(14-16)^.

Ademais, um estudo recente^(17)^ indica que a relação entre percepção e produção pode variar conforme a classe fonológica analisada, com correlação significativa entre produção e percepção observada apenas na classe das fricativas.

Considerando a necessidade de explorar a relação entre produção e percepção de fala no processo terapêutico de crianças com TF, este estudo tem como objetivo comparar e correlacionar as acurácias de percepção no outro, percepção em si e produção de fala em crianças com TF submetidas à intervenção fonoaudiológica para supressão do processo de substituição de líquidas.

A escolha do processo de substituição de líquidas se justifica por se tratar de um processo fonológico de aquisição tardia, que demanda maior refinamento das habilidades de percepção e produção devido à sua complexidade acústico-articulatória^(18)^. Além disso, trata-se de um processo frequentemente persistente em crianças com TF, com impacto significativo na inteligibilidade da fala. Dessa forma, seu estudo é relevante tanto do ponto de vista clínico, para orientar intervenções mais eficazes, quanto teórico, para aprofundar a compreensão dos padrões fonológicos típicos e atípicos do português brasileiro.

As hipóteses do estudo são: (1) espera-se encontrar diferenças entre as habilidades de percepção (no outro e em si) e produção ao longo do processo de intervenção; e (2) espera-se observar correlação positiva entre as habilidades de percepção e produção.

## MÉTODO

### Amostra selecionada

Após aprovação do Comitê de Ética em Pesquisa (CEP), sob o número 30672720.3.0000.5406, foram selecionadas, por conveniência, duas crianças provenientes das listas de espera de unidades básicas de saúde e 14 crianças oriundas de escolas municipais e estaduais da Educação Infantil e do Ensino Fundamental I, localizadas próximas ao local em que seria realizada a intervenção.

Os critérios de inclusão foram: (a) crianças monolíngues entre quatro e oito anos (média de idade 5,9 anos); (b) crianças com diagnóstico de TF que apresentavam, necessariamente, mas não exclusivamente, o processo de substituição de líquidas (/ɾ/ → [l] ou /l/ → [ɾ])^(18),^ conforme a Avaliação Fonológica da Criança (AFC); e (c) disponibilidade dos responsáveis e da criança para participar das sessões de intervenção.

Optou-se por incluir crianças a partir dos quatro anos, uma vez que o diagnóstico de TF pode ser realizado a partir dos três anos, quando já é possível identificar processos fonológicos não esperados para a idade. Além disso, crianças com TF frequentemente apresentam múltiplos processos de simplificação da fala, o que pode aumentar o risco de atrasos na aquisição de outros fonemas^(19)^.

Os critérios de exclusão consistiram na presença de: (a) alterações estruturais dos órgãos fonoarticulatórios (ex.: anquiloglossia, respiração oral e entre outros); (b) diagnóstico de Transtorno da Linguagem associado ao TF^(1)^ – cabe esclarecer que o sistema de classificação diagnóstica adotado no sistema público de saúde advém de uma perspectiva médica, apesar de ambos os diagnósticos envolverem alterações linguísticas que podem se fazer presentes no desenvolvimento infantil –; (c) comorbidades que afetasse a produção da fala; (d) queixa ou alterações auditivas, como limiares superiores a 15 dBNA nas frequências de 500, 1K, 2K e 4K; (e) síndromes ou transtornos do neurodesenvolvimento e (f) não adesão ao programa ou possível desistência durante a intervenção.

Após a seleção e a assinatura do Termo de Consentimento Livre e Esclarecido (TCLE), os participantes foram submetidos à: anamnese fonoaudiológica, avaliação da linguagem durante o brincar, exame das estruturas orofaciais, avaliação audiológica básica e testes específicos de fala, com o uso do Instrumento de Avaliação de Fala para Análise Acústica – IAFAC^(20)^ composto por 28 palavras com fonemas na vogal /a/ em posição acentuada.

As amostras de fala foram obtidas por repetição e/ou nomeação espontânea de imagens e gravadas para análise perceptivo-auditiva por, no mínimo, dois juízes. A análise incluiu transcrição fonética, levantamento do inventário fonético, variabilidade de produção, caracterização do sistema fonológico e cálculo do índice de gravidade do TF, por meio do Percentual de Consoantes Corretas - revisado (PCC-r)^(21)^.

Embora a repetição e a nomeação envolvam diferentes processos psicolinguísticos, crianças com TF frequentemente evitam a nomeação de palavras, especialmente durante a terapia devido à complexidade das produções. Nesses casos, os terapeutas incentivaram a repetição, mesmo que facilitada pelo modelo adulto.

A Tabela 1, abaixo, apresenta a caracterização dos participantes selecionados para o estudo quanto a idade, sexo (“M” - masculino e “F” - feminino) e índice de gravidade do TF (PCC-r)^(21)^.

**Tabela 1:** Caracterização dos sujeitos

Sujeito	Idade	Sexo	PCC-r (%)	Gravidade
S1	6:5 anos	M	89,7	Leve
S2	5:11 anos	M	87,1	Leve
S3	4:11 anos	F	77,6	Levemente-moderado
S4	7:3 anos	F	91,5	Leve
S5	6:11 anos	M	92,2	Leve
S6	5:5 anos	M	94,2	Leve
S7	4:5 anos	F	62,9	Moderadamente-severo
S8	5:0 anos	M	83,5	Levemente-moderado
S9	6:11 anos	M	57,3	Moderadamente-severo
S10	6:2 anos	F	82,9	Levemente-moderado
S11	7:0 anos	M	85,3	Leve
S12	8:9 anos	F	82,6	Levemente-moderado
S13	6:3 anos	M	59,0	Moderadamente-severo
S14	4:8 anos	M	67,4	Levemente-moderado
S15	4:3 anos	F	73,4	Levemente-moderado
S16	6:7 anos	F	80	Leve

**Legenda:** “PCC-r” = Percentual de Consoantes Corretas-revisado; “S” = Sujeito; “M” = Masculino; “F” = Feminino. Fonte: elaborado pelos autores

O Quadro 1 apresenta o perfil fonético-fonológico das crianças, incluindo o inventário fonético (capacidade de produção dos sons da fala), o sistema fonológico (desenvolvimento da representação dos sons da fala)^(19)^ e os processos fonológicos.

**Quadro 1:** Perfil fonético-fonológico dos participantes

Sujeito	Inventário fonético – consoantes	Sistema fonológico – efetivamente adquiridos	Sistema fonológico – fonemas trabalhados	Processo fonológico trabalhado	Outros processos fonológicos envolvidos
S1	[p, b, t, d, k, g, v, f, s, z, ʒ, ʃ, m, n, ɲ, l, ɾ, x, ɹ]	/p, b, t, d, k, g, v, f, s, z, ʒ, ʃ, m, n, ɲ, x, ɾ, ɹ/	/l/ - não adquirido *(50% de acerto)* /ɾ/ - efetivamente adquirido *(100% de acerto)*	Substituição de líquida lateral para não-lateral	Semivocalização de líquida lateral e redução de encontro consonantal
S2	[p, b, t, d, k, g, v, f, s, z, ʒ, ʃ, n, ɲ, l, ɾ, x]	/p, b, t, d, k, g, v, f, s, z, ʒ, ʃ, n, ɲ, x/	/l/ - não adquirido *(50% de acerto)* /ɾ/ - não adquirido *(0% de acerto)*	Substituição de líquida não-lateral por lateral	Posteriorização de nasal, semivocalização de líquida lateral, apagamento de coda retroflexa e redução de encontro consonantal
S3	[p, b, t, d, k, g, f, v, s, n, l, x]	/p, t, k, g, s, f, v, s, n, l, x/	/l/ - efetivamente adquirido *(100% de acerto)* /ɾ/ - não adquirido *(0% de acerto)*	Substituição de líquida não-lateral por lateral	Dessonorização de obstruinte, anteriorização de fricativa, substituição de nasal bilabial por lateral alveolar, apagamento de nasal palatal, apagamento de coda fricativa e retroflexa, semivocalização de líquida lateral palatal e redução de encontro consonantal.
S4	[p, b, t, d, k, g, v, f, s, z, ʒ, ʃ, m, n, ɲ, l, ʎ, x, ɹ]	/p, b, t, d, k, g, v, f, s, z, ʒ, ʃ, m, n, ɲ, l, x, ɹ/	/l/ - efetivamente adquirido *(100% de acerto)* /ɾ/ - não adquirido *(0% de acerto)*	Substituição de líquida não-lateral por lateral	Redução e substituição de encontro consonantal.
S5	[p, b, t, d, k, g, v, f, s, z, ʒ, ʃ, m, n, ɲ, l, ʎ, ɾ, x, ɹ]	/p, b, t, d, k, g, v, s, z, ʒ, ʃ, m, n, ɲ, l, ʎ, x, ɹ/	/l/ - efetivamente adquirido *(100% de acerto)* /ɾ/ - não adquirido *(0% de acerto)*	Substituição de líquida não-lateral por lateral e lateral por não-lateral	Substituição de encontro consonantal não-lateral para lateral.
S6	[p, b, t, d, k, g, v, f, s, z, ʒ, ʃ, m, n, ɲ, l, ʎ, x, ɹ]	/p, b, t, d, k, g, v, f, s, z, ʒ, ʃ, m, n, ɲ, l, ʎ, x, ɹ/	/l/ - efetivamente adquirido *(100% de acerto)* /ɾ/ - não adquirido *(0% de acerto)*	Substituição de líquida não-lateral por lateral	Redução e substituição de encontro consonantal.
S7	[p, b, t, d, k, g, f, v, s, ʃ, ʒ, m, n, ɲ, l, x]	/p, b, t, d, v, s, ʃ, ʒ, m, n, ɲ, l, x/	/l/ - efetivamente adquirido *(100% de acerto)* /ɾ/ - não adquirido *(0% de acerto)*	Substituição de líquida não-lateral por lateral	Anteriorização de oclusiva, posteriorização de fricativa, substituição de líquida lateral por alveolar, semivocalização de coda retroflexa e substituição de encontro consonantal não-lateral por lateral.
S8	[p, b, t, d, k, g, f, v, s, z, ʒ, m, n, ɲ, l, ɾ, x]	/p, b, t, d, k, g, f, v, s, z, ʒ, m, n, ɲ, x/	/l/ - não adquirido *(33,3% de acerto)* /ɾ/ - em concorrência *(55,5% de acerto)*	Substituição de líquida não-lateral por lateral e lateral por não-lateral	Anteriorização de fricativa, apagamento e semivocalização de líquida, apagamento de coda retroflexa e redução de encontro consonantal.
S9	[p, b, t, k, f, ʃ, ʒ, m, n, ɲ, l, ɾ, x]	/p, t, k, ʃ, m, n, ɲ, x/	/l/ - não adquirido *(50% de acerto)* /ɾ/ - não adquirido *(12% de acerto)*	Substituição de líquida não-lateral por lateral e lateral por não-lateral	Dessonorização de obstruinte, plosivização e posteriorização de fricativa, apagamento de coda semivocálica, retroflexa e nasal, substituição de líquida lateral e redução de encontro consonantal.
S10	[p, b, t, d, k, g, f, v, s, z, m, n, ɲ, l, ʎ, x]	/p, b, t, d, k, g, f, v, s, z, m, n, ɲ, l, ʎ, x/	/l/ - efetivamente adquirido *(100% de acerto)* /ɾ/ - não adquirido *(0% de acerto)*	Substituição de líquida não-lateral por lateral	Anteriorização de fricativa, semivocalização de coda retroflexa, redução de encontro consonantal.
S11	[p, b, t, d, k, g, f, v, s, z, ʃ, ʒ, m, n, ɲ, l, ʎ, x, ɹ]	/p, b, t, d, k, g, f, v, s, z, ʃ, ʒ, m, n, ɲ, l, ʎ, x, ɹ/	/l/ - efetivamente adquirido *(100% de acerto)* /ɾ/ - não adquirido *(0% de acerto)*	Substituição de líquida não-lateral por lateral	Redução de encontro consonantal.
S12	[p, b, t, d, k, g, f, v, s, z, ʃ, ʒ, m, n, ɲ, l, ʎ, x]	/p, b, t, d, k, g, f, v, s, z, ʃ, ʒ, m, n, ɲ, l, ʎ, x/	/l/ - efetivamente adquirido *(100% de acerto)* /ɾ/ - não adquirido *(0% de acerto)*	Substituição de líquida não-lateral por lateral	Apagamento de coda retroflexa e redução de encontro consonantal.
S13	[p, b, t, k, g, f, v, s, ʒ, m, ɲ, l, x]	/p, b, k, g, f, v, m, ɲ, x/	/l/ - não adquirido *(50% de acerto)* /ɾ/ - não adquirido *(0% de acerto)*	Substituição de líquida não-lateral por lateral	Posteriorização de obstruinte e nasal alveolar, apagamento e de líquida lateral e não-lateral, semivocalização de líquida lateral palatal, apagamento de coda retroflexa e redução de encontro consonantal.
S14	[p, b, t, d, k, g, f, v, s, ʃ, m, n, ɲ, l, x]	/p, b, d, k, g, s, n, ɲ, l, x/	/l/ - efetivamente adquirido *(100% de acerto)* /ɾ/ - não adquirido *(0% de acerto)*	Substituição de líquida não-lateral por lateral	Posteriorização de obstruinte, substituição de fricativa lábio-dental por alveolar, substituição de nasal bilabial por líquida lateral, dessonorização de fricativa, plosivização de fricativa alveolar por oclusiva velar, substituição de líquida palatal por dentoalveolar, apagamento de coda fricativa e retroflexa, substituição de plosiva por fricativa, metátese em encontro consonantal e redução de encontro consonantal.
S15	[p, b, t, d, k, g, f, v, s, ʃ, m, n, ɲ, l, ʎ, ɾ, x, ɹ]	/p, b, t, d, k, g, f, ʃ, m, n, ɲ, l, ʎ, x/	/l/ - efetivamente adquirido *(100% de acerto)* /ɾ/ - não adquirido *(0% de acerto)*	Substituição de líquida não-lateral por lateral	Plosivização, anteriorização e dessonorização de fricativa, semivocalização de coda retroflexa e redução de encontro consonantal.
S16	[p, b, t, d, k, g, f, v, s, z, ʃ, ʒ, m, n, ɲ, l, ʎ, x, ɹ]	/p, b, t, d, f, v, s, z, ʃ, ʒ, m, n, ɲ, l, ʎ, x, ɹ /	/l/ - efetivamente adquirido *(100% de acerto)* /ɾ/ - não adquirido *(30% de acerto)*	Substituição de líquida não-lateral por lateral	Plosivização e anteriorização de fricativa, semivocalização de coda retroflexa, dessonorização de fricativa e redução de encontro consonantal.

Fonte: elaborado pelos autores

### Programa de intervenção

O programa de intervenção fonológica adotado baseou-se no “modelo de percepção-produção” (Berti, em preparação), atualmente em fase de elaboração e validação. Esse modelo foi escolhido por ser, até o momento, o único a abordar simultaneamente a percepção da fala do terapeuta (percepção no outro), a percepção da própria fala da criança (percepção em si) e a produção da fala.

O processo de intervenção consistiu em 16 sessões fonoaudiológicas, com foco no processo fonológico de substituição de líquidas (/l/ → [ɾ] ou /ɾ/ → [l]), realizadas duas vezes por semana (às terças e sextas-feiras), com duração de 50 minutos cada, totalizando 13 horas e 30 minutos de atendimentos.

O número de sessões foi definido com base na literatura, que recomenda um mínimo de 15 atendimentos de terapia^(2)^. Contudo, as crianças poderiam concluir o tratamento antes da 16ª sessão caso superassem o processo fonológico, ou continuar em atendimento no serviço de saúde, se ainda necessitassem de intervenção.

Foram selecionadas 30 palavras-alvo (com os sons-alvos de /l/ e /ɾ/ na posição de *onset* simples) para serem trabalhadas em todas as etapas da intervenção, e outras 30 palavras-sondagem (com os sons-alvo de /l/ e /ɾ/ em *onset* simples), utilizadas exclusivamente para avaliar a generalização. A maioria das palavras formava pares mínimos ou pares análogos, conforme apresentado na Tabela 2.

**Tabela 2:** Palavras selecionadas para a intervenção

Palavras-alvo (PA)		Palavras-sondagem (PS)	
/ɾ/	/l/	/ɾ/	/l/
Vera	Vela	Vareta	Valeta
Mara	Mala	Carango	Calango
Caro	Calo	Barão	Balão
Puro	Pulo	Pário	Palio
Cara	Cala	Caruso	Caluso
Sara	Sala	Peru	Pelu
Vira	Vila	Gari	Gali
Vara	Vala	Poro	Pólo
Corado	Colado	Rara	Rala
Cera	Sela	Garo	Galo
Mora	Mola	Gera	Pala
Marinha	Malinha	Farinha	Bala
Mira	Mila	Jararaca	Cola
Sarada	Salada	Maracá	Bela
Coragem	Colagem	Pera	Pistola

Fonte: elaborado pelos autores

O processo de intervenção consistiu em seis etapas:

1. Avaliação inicial - Coleta e análise da amostra de fala por meio do IAFAC^(20)^, composto por 28 palavras com fonemas na vogal /a/ em posição acentuada, para seleção dos participantes e início da intervenção.
2. Pré-terapia – Coleta inicial com gravação em áudio da nomeação espontânea ou repetição das 30 palavras-alvo e 30 palavras-sondagem. As palavras foram apresentadas por meio de imagens em computador, contextualizadas com atividades lúdicas e brinquedos de interesse da criança. As produções foram gravadas com mínima interferência da terapeuta.
3. Explicação do processo fonológico – Apresentação contrastiva dos sons-alvos (/l/ e /ɾ/) utilizando materiais lúdicos (ex.: representação do bocão e da língua, construídos com itens recicláveis) para demonstrar os movimentos da língua (elevação e batida rápida da língua na produção do [ɾ] e elevação e batida lenta da língua na produção do [l]), sendo exemplificado com palavras-alvo em pares mínimos, mostrando para a criança como são os movimentos e se os substituirmos, o significado da palavra também é alterado (ex.: [´kaɾu] — algo que custa muito dinheiro – e [´kalu] – machucado das mãos ou dos pés após muito esforço).
4. Percepção no outro – As 30 palavras-alvo foram produzidas pela terapeuta, de forma ao vivo e natural, solicitando-se que as crianças identificassem os sons alvos: [l] e [ɾ]. Por exemplo, durante o momento de um jogo do interesse da criança, uma palavra-alvo era sorteada, a terapeuta se posicionava na frente e na altura da criança – atraindo a total atenção da criança para o seu rosto –, produzia a palavra de maneira devagar e com ênfase no som-alvo para, em seguida, a criança responder qual dos sons alvos foi produzido. Cada resposta da criança era anotada como acerto ou erro.
5. Percepção em si – As crianças produziam as 30 palavras-alvo e, em seguida, relatavam qual dos sons alvos havia produzido ([l] e [ɾ]). Pistas auditivas, visuais e/ou proprioceptivas eram fornecidas conforme a necessidade. As respostas também foram classificadas como acerto ou erro.
6. Produção – As crianças produziam as 30 palavras-alvo. Quando necessário, foram utilizadas pistas visuais (ex.: uso de espelho) e táteis-cinestésicas (ex.: produção repetitiva dos fones [d] ou [l] para propiciar a produção alvo), permanecendo em contexto lúdico e motivador à criança. As produções foram registradas como acerto, erro ou gradiência (realizações intermediárias entre erro e acerto).
7. Pós-terapia – Coleta final com reaplicação do IAFAC^(20)^ e nova gravação da nomeação ou repetição das 30 palavras-alvo e 30 palavras-sondagem, nas mesmas condições da coleta inicial.

Entre as etapas de percepção no outro, percepção em si e produção de fala, sempre era realizada uma sondagem com as palavras não trabalhadas na terapia. Caso a criança atingisse 80% de acertos, avançava-se para próxima etapa. Esse critério seguiu os parâmetros de Yavas et al.^(17)^, que consideram um som contrastivo como adquirido entre 76% e 85% de acerto.

O esquema da intervenção pode ser visualizado na Figura 1

**Figura 1:** Modelo de intervenção fonoaudiológica proposto

Durante todas as etapas, a intervenção foi conduzida por duas fonoaudiólogas formadas e mestrandas em Fonoaudiologia, com a colaboração de uma estudante da graduação em Fonoaudiologia devidamente supervisionada.

Ao final de cada sessão, foram realizadas gravações das produções das palavras-alvo e das palavras-sondagem, totalizando 512 gravações (uma gravação de produção de palavras-alvo + uma gravação de produção de palavras-sondagem x 16 sessões de atendimento do programa de intervenção x 16 crianças participantes).

As produções foram avaliadas por dois juízes fonoaudiólogos, que classificaram os sons como: (1) produção correta, (2) produção incorreta ou (3) produção gradiente. Em caso de discordância, um terceiro juiz fonoaudiólogo foi acionado para desempate. Posteriormente, foi calculado o percentual de acerto ao longo das sessões.

### Análise estatística

Para análise estatística inferencial considerou-se a acurácia das crianças em seis momentos terapêuticos: 1) produção de palavras-alvo pré-terapia; 2) produção de palavras-sondagem pré-terapia; 3) percepção no outro na primeira sessão; 4) percepção em si na primeira sessão; 5) produção de palavras-alvo pós-terapia; e 6) produção de palavras-sondagem pós-terapia.

Para a análise comparativa utilizou-se o desempenho percentual (de 0 a 100% de acerto) em cada uma das etapas. Já para a análise de correlação, foram utilizadas as respostas individuais por palavra (30 palavras-alvo e 30 palavras sondagem), caracterizadas como: (1) acerto e (0) erro, tanto para percepção quanto para produção de fala.

Na comparação dos desempenhos, utilizou-se a ANOVA de Medidas Repetidas, seguida do teste de post-hoc de Tukey para identificar diferenças entre as habilidades.

Para a análise de correlação, utilizou-se o teste não paramétrico de correlação de Spearman, que avalia a força da associação entre variáveis, com coeficientes variando de -1 a +1 (sendo 0 indicativo de ausência de correlação e ±1 indicativo de correlação perfeita). O nível de significância adotado foi de α < 0,05.

## RESULTADOS

A Tabela 3 apresenta a acurácia (média percentual de acertos e desvio-padrão) nas habilidades analisadas ao longo da intervenção, considerando os seguintes momentos: produção de palavras-alvo (pré-terapia); produção de palavras-sondagem (pré-terapia); primeira sessão de percepção no outro; primeira sessão de percepção em si; produção de palavras-alvo (pós-terapia) e produção de palavras-sondagem (pós-terapia).

**Tabela 3:** Porcentagem média de acertos das habilidades trabalhadas no processo de intervenção por sujeito

Sujeito	Produção de PA pré-terapia	Produção de PS pré-terapia	1º sessão PO	1º sessão PSi	Produção de PA pós-terapia	Produção de PS pós-terapia
S1	60	50	96,6	100	96,6	90
S2	50	59,2	100	70	100	80
S3	50	50	94,7	93,3	50	60
S4	53,3	50	80	93,3	50	60
S5	63,3	70	100	96,6	96,6	93,3
S6	63,3	70	100	96,6	96,6	90
S7	50	50	89,6	96,6	50	56,6
S8	66,6	53,3	80	83,3	56,6	56,6
S9	70	70	86,6	83,3	76,6	63,3
S10	50	50	76,6	93,3	96,6	76,6
S11	66,6	60	96,6	100	83,3	76,6
S12	56,6	53,3	100	100	83,3	86,6
S13	23,3	20	100	100	20	36,6
S14	60	50	93,3	83,3	63,3	63,3
S15	50	53,3	90	100	75,8	83,3
S16	86,6	60	96,6	93,3	100	90
Média Geral	57,5	54,3	92,5	92,7	74,7	72,7
Desvio Padrão	13,46	11,89	7,97	8,54	23,99	16,40

**Legenda:** “PA” = Palavras-alvo; “PS” = palavras-sondagem; “PO” = percepção no outro; ”PSi” = percepção em si; “S” = sujeito. Fonte: elaborado pelos autores

A Tabela 4 mostra os resultados da ANOVA de medidas repetidas que indicou diferença estatisticamente significativa entre as habilidades trabalhadas (F(5,25) = 29,87, p < 0.01). A partir do teste pos hoc de Tukey, observou-se diferença entre as habilidades de produção de palavras-alvo e palavras sondagem no início e ao término da intervenção; como também se observou diferença entre as habilidades de percepção (no outro e em si) e as habilidades de produção de fala (palavras-alvo e sondagem).

**Tabela 4:** Comparação entre as habilidades (ANOVA de Medidas Repetidas)

HABILIDADES	Produção PA pré-terapia	Produção PS pré-terapia	1º sessão Percepção no outro	1º sessão Percepção em si	Produção PA pós-terapia	Produção PS pós-terapia
Produção PA pré-terapia		0,9617	0,0001*	0,0001*	0,0002*	**0,0009** *
Produção PS pré-terapia	-		0,0001*	0,0001*	0,0001*	0,0001*
1º sessão Percepção no outro	**-**	**-**		1,0000	0,0134*	0,0023*
1º sessão Percepção em Si	**-**	**-**	-		0,0016*	0,0028*
Produção PA pós-terapia	**-**	**-**		**-**		0,9929
Produção PS pós-terapia	**-**	**-**	**-**	**-**		

* valores em negrito = p<0,05

**Legenda:** “PA” = Palavras-alvo; “PS” = palavras-sondagem. Fonte: elaborado pelos autores

Não foram verificadas diferenças entre a percepção no outro e percepção em si, nem entre a produção de palavras-alvo e palavras-sondagem no momento pré-terapia.

A Figura 2 ilustra a acurácia das crianças ao longo das etapas do processo terapêutico.

**Figura 2:** Ilustração do efeito entre as habilidades (ANOVA de Medidas Repetidas)

**Legenda:** “PROD” = produção; “PA-pré” = palavras-alvo pré-terapia; “PS-pré” = palavras-sondagem pré-terapia; “PO” = percepção no outro; “PSI” = percepção em si; “PA-pós” = palavras-alvo pós-terapia”; “PS-pós” = palavras sondagem pós-terapia.

Fonte: elaborado pelos autores

A Tabela 5 apresenta os dados de correlação entre as habilidades de percepção no outro, percepção em si, produção das palavras-alvo e produção das palavras sondagem.

**Tabela 5:** Direção e força de correlação entre as habilidades (valor de r) (Teste de correlação de Spearman)

Variáveis	Produção PA pré-terapia	Produção PS pré-terapia	1º sessão Percepção no outro	1º sessão Percepção em si	Produção PA pós-terapia	Produção PS pós-terapia
Produção PA pré-terapia		0,182*	0,036	-0,039	**0,280** *	0,078
Produção PS pré-terapia	**-**		-0,008	-0,030	-0,082	0,377*
1º sessão Percepção no outro	-	-		0,034	0,119*	0,059
1º sessão Percepção em si	-	-	-		0,085*	0,106*
Produção PA pós-terapia	**-**	-	**-**	**-**		0,269*
Produção PS pós-terapia	-	**-**	-	**-**	**-**	

* valores em negrito = p<0,05

**Legenda:** “PA” = Palavras-alvos; “PS” = palavras-sondagem. Fonte: elaborado pelos autores

Foram identificadas correlações positivas entre percepção no outro e produção de palavras-alvo pós-terapia (r = 0,11), percepção em si e produção de palavras-alvo (r = 0,08), percepção em si e produção de palavras-sondagem pós-terapia (r = 0,10).

A correlação mais forte se deu entre percepção no outro e produção de palavras-alvo, sugerindo que o desempenho perceptivo inicial pode influenciar diretamente a melhora na produção de palavras utilizadas em terapia.

A fim de ilustrar as generalizações observadas ao final da intervenção, o Quadro 2 apresenta o percentual de acertos nas palavras-sondagem no momento pós-terapia, por participante. Nota-se que os sujeitos com TF leve apresentaram, em geral, maiores índices de acertos nas palavras não trabalhadas, sugerindo melhor generalização. Já entre os sujeitos com TF de gravidade moderada a severa, os percentuais de acerto foram, em sua maioria, mais baixos, o que indica uma possível relação entre a gravidade do transtorno e a capacidade de generalização para contextos não trabalhados.

**Quadro 2:** Percentual de acertos nas palavras-sondagem ao final da intervenção (indicador de generalização)

Sujeito	PCC-r (%)	Gravidade do TF	Acerto nas PS após a terapia (%)
S1	89,7	Leve	90
S2	87,1	Leve	80
S3	77,6	Levemente moderado	60
S4	91,5	Leve	60
S5	92,2	Leve	93,3
S6	94,2	Leve	90
S7	62,9	Moderadamente severo	56,6
S8	83,5	Levemente moderado	56,6
S9	57,3	Moderadamente severo	63,3
S10	82,9	Levemente moderado	76,6
S11	85,3	Leve	76,6
S12	82,6	Levemente moderado	96,6
S13	59,0	Moderadamente severo	36,6
S14	67,4	Levemente moderado	63,3
S15	73,4	Levemente moderado	83,3
S16	80	Leve	90

**Legenda:** PS = palavras-sondagem; PCC-r = Percentual de Consoantes Corretas - revisado;

TF = Transtorno Fonológico. Fonte: elaborado com base na tabela dois deste estudo

Em síntese, os resultados demonstram que as 16 crianças apresentaram desempenhos distintos ao longo da intervenção, refletindo a heterogeneidade clínica do TF.

Na comparação entre as habilidades, verificou-se que percepção e produção diferiram entre si, enquanto as correlações encontradas sugerem que o aprimoramento perceptivo pode influenciar positivamente a produção de fala. Ademais, os resultados de acerto na produção das palavras-sondagem ao final da intervenção evidenciam que a percepção em si pode estar relacionada à generalização dos sons-alvo, com melhor desempenho observado entre crianças com TF leve.

## DISCUSSÃO

O objetivo deste estudo foi comparar e correlacionar as acurácias de percepção no outro, percepção em si e produção de fala em crianças com TF submetidas à intervenção fonoaudiológica.

A primeira hipótese previa que as habilidades de percepção e produção apresentariam desempenhos distintos durante a intervenção. De fato, ao comparar as acurácias dos desempenhos nas habilidades de percepção no outro, percepção em si e produção de palavras-alvo e palavras-sondagem, tanto nos momentos pré quanto pós-terapia, foram observadas diferenças estatisticamente significativas, confirmando essa hipótese. Contudo, não houve diferença significativa entre as habilidades de percepção no outro e percepção em si, sugerindo semelhança entre elas.

Essa semelhança pode ser explicada pelo fato de ambas as habilidades envolverem a conscientização dos sons-alvos (/l/ e /ɾ/) por parte da criança^(21)^. No entanto, a percepção em si tem sido pouco explorada na literatura, apesar de ser reconhecida como uma habilidade crucial para a aquisição fonológica^(19-22)^.

Diversos modelos terapêuticos ^(9-13)^ priorizam a percepção auditiva a partir da fala do adulto típico, em detrimento à escuta ativa da própria fala da criança. Até o momento, não foram encontrados estudos que comparam diretamente a percepção no outro e em si durante a terapia, embora estudo como o de Battistela^(23),^ já tenha destacado a sua relevância.

Os resultados também indicam que a percepção e a produção se comportam de maneira distinta ao longo do processo terapêutico, o que reforça achados de estudos anteriores, a saber: uma boa percepção nem sempre se traduz em uma produção adequada^(5-7)^.

No presente estudo, sete das 16 crianças não adquiriram os fonemas-alvos ao final da intervenção, o que pode ser justificado devido à menor idade, maior número de processos fonológicos envolvidos e/ou inventário fonético mais restrito.

O salto quantitativo observado na acurácia das produções de palavras-alvo e palavras-sondagem no momento pré-terapia e na acurácia da percepção no outro e percepção em si pode ter sido influenciado pela etapa de explicação do processo fonológico. Nesta etapa, foram apresentados de forma contrastiva os sons-alvo (/l/ e /ɾ/), com uso de recursos visuais e lúdicos que explicitavam os movimentos articulatórios envolvidos em cada produção. Esse trabalho prévio parece ter contribuído para o aumento da consciência articulatória das crianças, favorecendo tanto o reconhecimento perceptivo dos sons quanto a identificação consciente de suas próprias produções, o que pode ter facilitado o desempenho nas etapas subsequentes de percepção e, indiretamente, nas de produção.

A diversidade de respostas entre crianças reforça o caráter heterogêneo do TF e evidencia a necessidade de controlar variáveis em futuros estudos como idade, gravidade do TF e perfil fonético-fonológico.

Adicionalmente, observando o desempenho das crianças segundo a gravidade do TF, conforme o índice PCC-r^(21)^, notou-se que as crianças classificadas com TF leve apresentaram, em geral, maior percentual de acertos nas etapas de percepção e produção em comparação àquelas com gravidade moderada ou severa. Esse dado sugere que a gravidade do transtorno pode influenciar a resposta à intervenção, especialmente no que se refere à generalização para palavras não trabalhadas. No entanto, devido ao tamanho limitado da amostra, essa análise deve ser interpretada com cautela e aprofundada em estudos futuros.

A segunda hipótese do estudo também foi confirmada, a qual previa-se a correlação positiva entre as habilidades de percepção e produção de fala. Observou-se correlação positiva entre a percepção no outro e a produção de palavras-alvo, bem como entre percepção em si e a produção de palavras-alvo e palavras-sondagem. Isso significa dizer que quanto melhor o desempenho da criança na etapa de percepção no outro, melhor será seu desempenho na produção de palavras trabalhadas em terapia. Além disso, quanto melhor o desempenho da criança na etapa de percepção em si, melhor o seu desempenho na produção tanto de palavras trabalhadas quanto não-trabalhadas em terapia, tornando a percepção em si uma habilidade extremamente importante para a generalização.

De fato, os dados do presente estudo revelaram que os percentuais de acertos nas palavras-sondagem ao final da intervenção reforçam esse achado: crianças com melhores desempenhos nas etapas de percepção em si também apresentaram maiores índices de generalização para palavras não trabalhadas.

Além disso, observou-se que os participantes com Transtorno Fonológico leve tiveram um desempenho melhor, obtendo mais acertos nas palavras. Isso sugere que a gravidade do transtorno pode influenciar a eficácia da generalização das habilidades. Esse resultado corrobora estudo anterior que mostrou uma relação entre o desempenho perceptivo, a severidade do Transtorno Fonológico e o sucesso na terapia em contextos que não foram especificamente treinados^(24)^.

As correlações entre percepção e produção reforçam a importância de trabalhar a percepção antes da produção da fala, tal como sugerido por abordagens fonológicas de intervenção^(9-13)^.

O fato de a correlação mais forte ocorrer entre percepção no outro e produção de palavras-alvo pós-terapia (r = 0,12), seguida pela correlação entre percepção em si e produção de palavras-sondagem (r = 0,10), revela que ambas as habilidades devem ser exploradas de forma complementar. A percepção da fala no outro envolve estímulos mais estáveis, com pistas mais acústicas mais robustas, o que pode facilitar o reconhecimento dos contrastes. Por outro lado, na percepção em si, a criança se baseia em suas próprias produções – muitas vezes ainda imprecisas^(16)^, o que torna essa tarefa mais desafiadora, mas potencialmente eficaz a longo prazo.

A relação entre percepção e produção é complexa e deve ser explorada em pesquisas com amostras maiores. Também recomenda-se considerar a gravidade do TF, aspectos socioeconômicos, aspectos socioeducacionais, e presença de outros processos fonológicos, o que pode contribuir para o entendimento da relação entre percepção e produção durante o processo terapêutico.

## CONCLUSÃO

Durante a intervenção fonoaudiológica, as crianças com TF apresentaram melhores desempenhos nas habilidades de percepção (no outro e em si) do que nas habilidades de produção de fala. Foram observadas correlações positivas entre percepção no outro e produção de palavras-alvo, bem como entre percepção em si e produção de palavras-alvo e palavras-sondagem.

Esses resultados indicam que o aprimoramento perceptivo pode influenciar positivamente o desempenho na produção. Particularmente, a percepção em si demonstrou estar associada a generalização de sons-alvos (/l/ e /ɾ/) para contextos trabalhados e não trabalhados em intervenção. Isso reforça a importância de incluir atividades voltadas à percepção da própria fala da criança nos planejamentos terapêuticos, promovendo um tratamento mais eficaz.
